# Sinonasal Carcinoma Presenting with Proptosis and Visual Impairment: A Case Report

**DOI:** 10.7759/cureus.63011

**Published:** 2024-06-24

**Authors:** Soumya Agrawal, KM Hiwale, Sunita Vagha

**Affiliations:** 1 Pathology, Jawaharlal Nehru Medical College, Datta Meghe Institute of Higher Education and Research, Wardha, IND

**Keywords:** multidisciplinary care, surgical excision, proptosis, headache, paranasal sinuses, sinonasal carcinoma

## Abstract

Sinonasal carcinoma is a rare but aggressive malignancy arising from the nasal cavity and paranasal sinuses. We present a case of a 40-year-old female who presented with a three-month history of headache, diminution of vision, and proptosis. Imaging studies revealed soft tissue density with bony erosion and extraconal extension in the left orbit. Histopathological examination confirmed sinonasal carcinoma with anaplastic changes. The patient underwent surgical excision of the tumor and received post-operative care in the neuro-ICU. Her visual acuity improved post-surgery, and she was discharged with stable neurological status. This case highlights the challenges in the diagnosis and management of sinonasal carcinoma and underscores the importance of multidisciplinary care for optimal outcomes. Early diagnosis and intervention are crucial in preventing complications and achieving favorable outcomes in patients with this aggressive malignancy.

## Introduction

Sinonasal carcinoma is a rare and aggressive malignancy originating in the nasal cavity and paranasal sinuses (PNS), accounting for less than 1% of all malignancies and around 3% of head and neck cancers. These tumors often present late due to their nonspecific symptoms, such as nasal obstruction, epistaxis, facial pain, and visual disturbances, which can mimic benign conditions such as chronic sinusitis [1,2]. The ethmoid sinuses are the most common site of origin, followed by the maxillary sinuses, nasal cavity, frontal sinuses, and sphenoid sinuses [3].

Advanced imaging techniques, including computed tomography (CT) and magnetic resonance imaging (MRI), play a crucial role in the diagnosis and staging of sinonasal carcinomas. CT scans are particularly useful for assessing bony involvement and the extent of soft tissue invasion, whereas MRI is superior for evaluating perineural spread and intracranial extension [4]. Histopathological examination remains the gold standard for definitive diagnosis, with various histological subtypes, such as squamous cell carcinoma, adenocarcinoma, and neuroendocrine carcinoma, influencing the treatment approach and prognosis [5].

Management of sinonasal carcinoma typically involves a combination of surgery, radiotherapy, and chemotherapy. Surgical resection aims to achieve clear margins, which is often challenging due to the proximity of critical anatomical structures such as the orbit and skull base. Postoperative radiotherapy is frequently employed to reduce the risk of local recurrence, and chemotherapy may be considered in advanced cases or for palliation [6]. Despite aggressive treatment, the prognosis remains poor, with a five-year survival rate ranging from 30% to 60% depending on the stage at diagnosis and the histological subtype [7]. This case report highlights a rare presentation of sinonasal carcinoma with significant orbital involvement in a 40-year-old female, emphasizing the importance of early detection and a multidisciplinary approach to management.

## Case presentation

A 40-year-old female presented to a tertiary care hospital in Central India with a three-month history of persistent headaches, progressive diminution of vision, and noticeable proptosis. These symptoms prompted a detailed evaluation in the neuro ward, where initial clinical findings included a visual acuity of 6/9 in the right eye and mere light perception in the left eye, which was also noted to have proptosis and ptosis.

**Figure 1:**
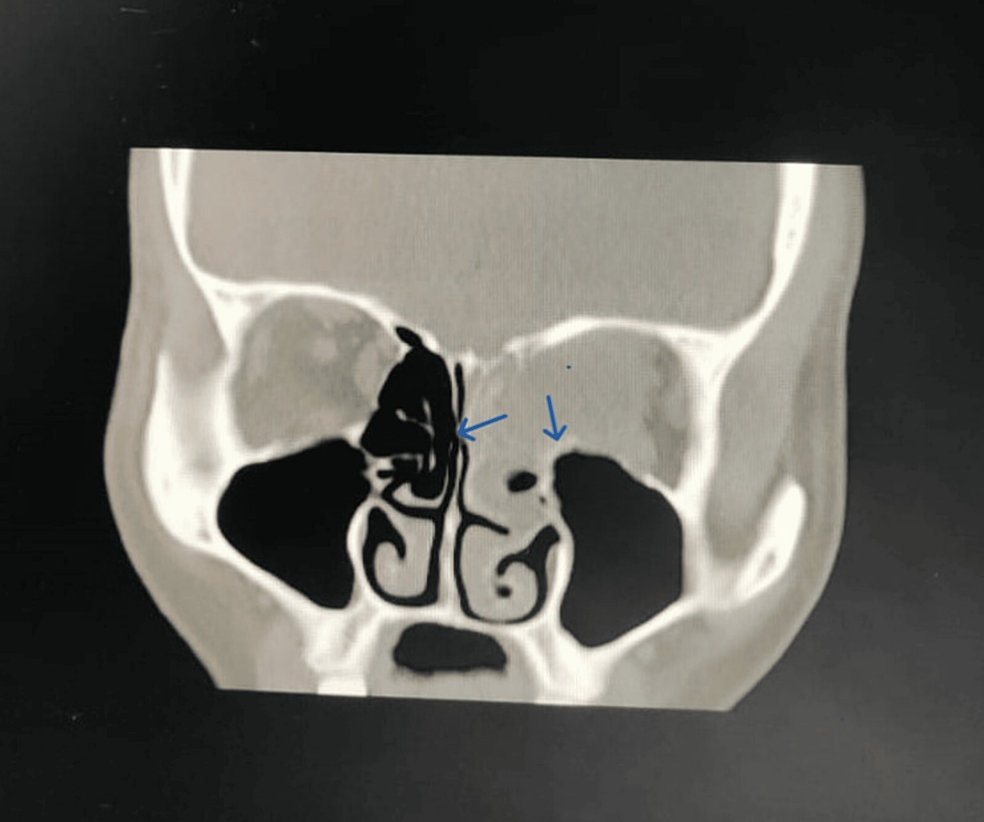
CT of the brain showing soft tissue attenuation mixed with air in the left ethmoid air cells, as well as bony destruction of the left lamina papyracea with a 22 x 10 mm extraconal soft tissue collection in the left orbit

A CT scan of the PNS revealed soft tissue density involving the left ethmoid and frontal sinuses, along with erosion of the left lamina papyracea and protrusion of the soft tissue. These findings indicated a significant pathological process. Further CT of the brain showed soft tissue attenuation mixed with air in the left ethmoid air cells, bony destruction of the left lamina papyracea with a 22 x 10 mm extraconal soft tissue collection in the left orbit (Figure 1), hyperdense collection in the left frontal sinus, mucosal thickening in the right ethmoid and bilateral maxillary sinuses, and scalp edema in the left parietal region. The imaging findings raised concerns about possible infective etiology, including rhino-ocular fungal disease.

**Figure 2:**
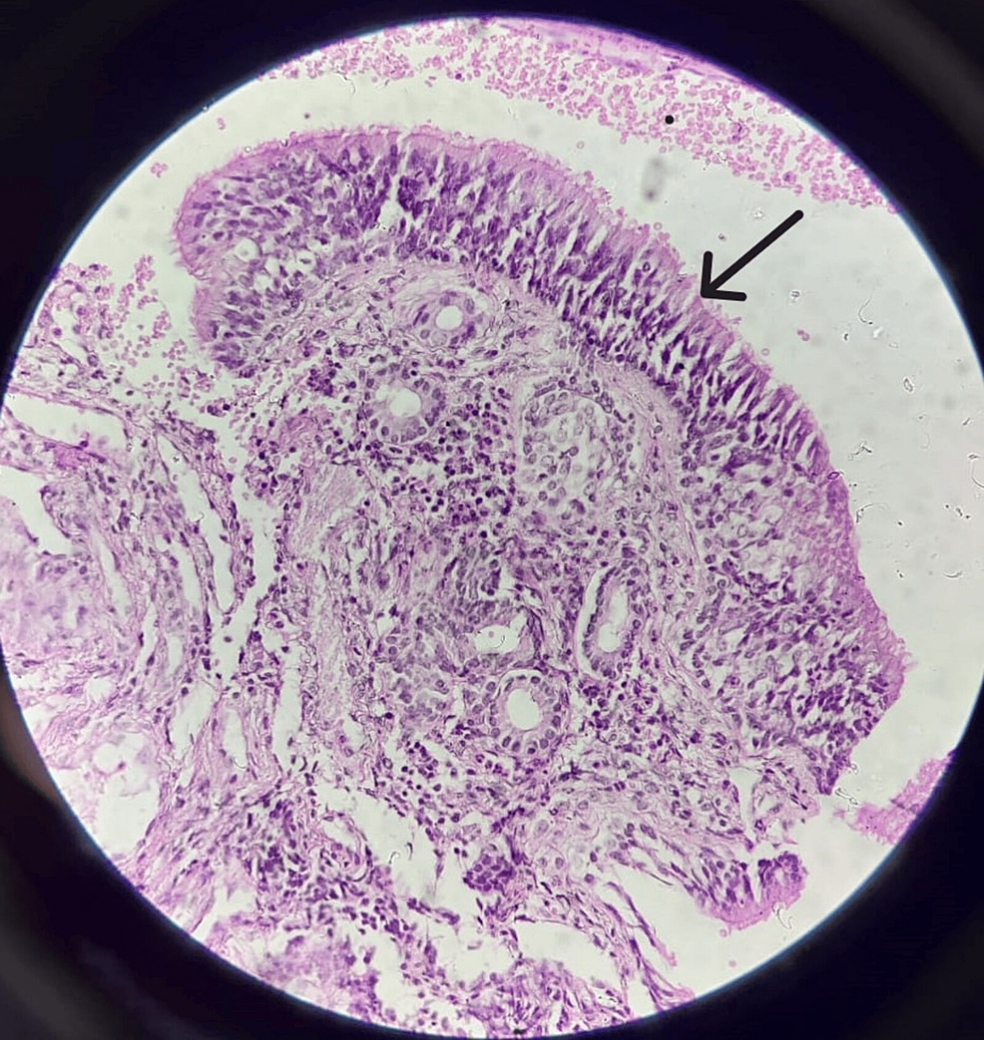
Section showing normal pseudostratified ciliated epithelium (20x)

**Figure 3:**
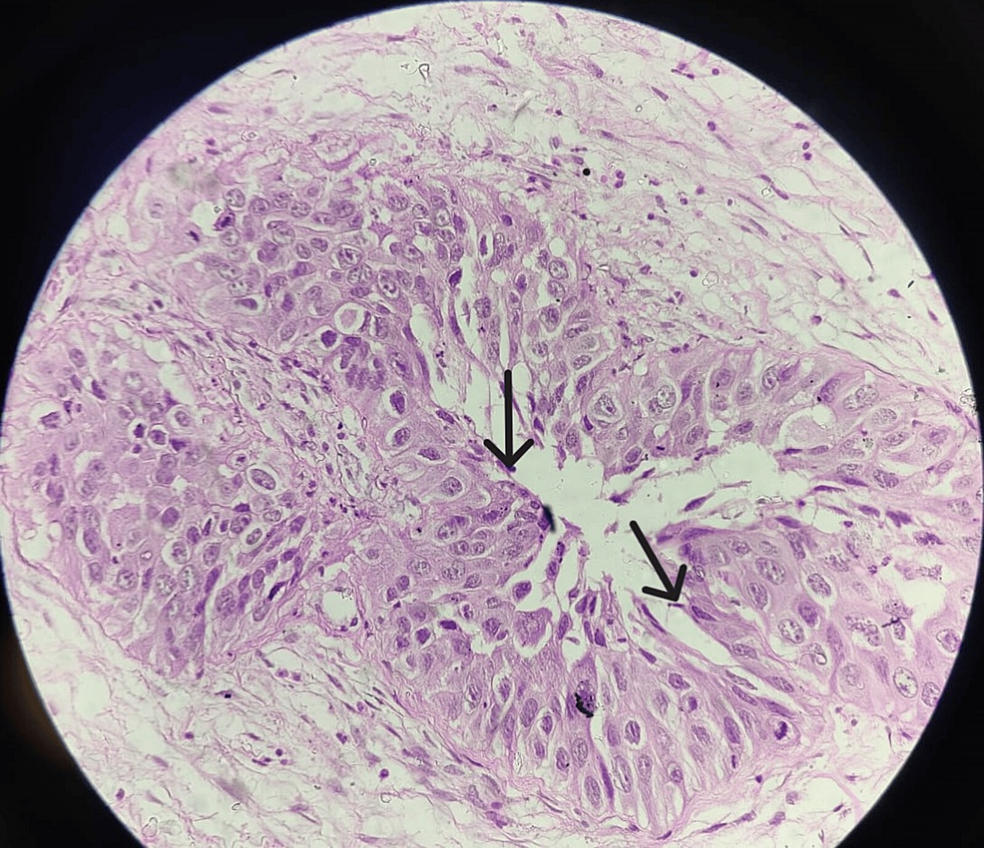
Section showing malignant anaplastic changes in the normal epithelium suggestive of sinonasal carcinoma (40x)

Histopathological examination of the excised tissue showed normal pseudostratified ciliated epithelium alongside malignant anaplastic changes, suggestive of sinonasal carcinoma. The cells exhibited scant cytoplasm with a basaloid appearance. Further examination at 40x magnification indicated densely populated small, monomorphic cells within a delicate fibrillar matrix with rounded nuclear contours, perinuclear clearing, and associated microcalcifications, resembling oligodendroglioma (Figures 2, 3).

Given these findings, the patient underwent surgical excision of a benign orbital tumor. Post-operatively, she was managed in the neuro-ICU with a regimen of antibiotics, analgesics, antacids, antiepileptics, and other supportive measures. Her postoperative course was uneventful, and her condition stabilized. Follow-up visual acuity assessment before discharge revealed improvement: the right eye measured 6/12 - 6/9 and the left eye improved to 6/12 - 6/6. The patient was neurologically and hemodynamically stable, conscious, and oriented at discharge. She was scheduled for a follow-up appointment in the neurosurgery outpatient department after 15 days to monitor her progress and manage any further treatment needs. This case underscores the importance of early detection and multidisciplinary management in patients with complex sinonasal pathologies, highlighting the potential for significant recovery with appropriate intervention.

## Discussion

Sinonasal carcinoma is a rare but aggressive malignancy arising from the epithelial cells of the nasal cavity and PNS. This case highlights the clinical presentation, diagnostic challenges, and therapeutic approach in managing a 40-year-old female with sinonasal carcinoma. The patient's presenting symptoms of headache, diminution of vision, and proptosis are consistent with advanced sinonasal malignancies, where the tumor invades surrounding structures such as the orbit and cranial cavity [8]. Imaging studies, including CT scans, are crucial for evaluating the extent of the disease. In this case, the CT of the PNS revealed soft tissue density with bony erosion and extraconal extension, indicative of an aggressive lesion [9]. Additionally, the findings of mucosal thickening and scalp edema pointed towards a potential infectious etiology, necessitating further histopathological confirmation.

Histopathological examination remains the gold standard for diagnosing sinonasal carcinoma. The presence of malignant anaplastic changes in the pseudostratified ciliated epithelium confirmed the diagnosis of sinonasal carcinoma, a finding that aligns with the literature on the histopathological features of this malignancy [10]. The identification of densely populated small, monomorphic cells with perinuclear clearing and microcalcifications further supported the diagnosis, mimicking oligodendroglioma-like features.

The management of sinonasal carcinoma typically involves a combination of surgical resection, radiotherapy, and chemotherapy, depending on the stage and extent of the disease [11]. In this case, the patient underwent surgical excision of the tumor, which is often the primary treatment modality aimed at complete resection to achieve local control [12]. Post-operative management in the neuro-ICU, including antibiotics and supportive care, was essential to prevent complications and ensure recovery.

The improvement in visual acuity post-surgery highlights the effectiveness of timely surgical intervention in preventing permanent ocular damage and restoring function [13]. The patient's stable neurological and hemodynamic status at discharge underscores the success of the multidisciplinary approach in managing such complex cases.

Early diagnosis and intervention are critical in sinonasal carcinoma due to its potential for rapid progression and invasion of adjacent structures [14]. This case underscores the importance of a comprehensive diagnostic workup, including advanced imaging and histopathological evaluation, to guide appropriate therapeutic strategies. Regular follow-up is essential to monitor for recurrence and manage any long-term sequelae.

## Conclusions

In conclusion, this case highlights the complexity of diagnosing and managing sinonasal carcinoma, emphasizing the significance of early detection, multidisciplinary collaboration, and meticulous post-operative care. The successful surgical excision of the tumor, coupled with appropriate adjuvant therapies and diligent monitoring, led to favorable outcomes, including improvement in visual acuity and stable neurological status. Moving forward, continued research efforts aimed at enhancing diagnostic modalities and treatment strategies are warranted to further improve outcomes and quality of life for patients with this aggressive malignancy.
